# Gesture production at encoding supports narrative recall

**DOI:** 10.1007/s00426-023-01886-w

**Published:** 2023-10-19

**Authors:** Naomi Sweller, Alexander-Jaehyuk Choi, Elizabeth Austin

**Affiliations:** 1 School of Psychological Sciences, Macquarie University, Sydney, NSW 2109 Australia; 2 Australian Institute of Health Innovation, Macquarie University, Sydney, NSW Australia

## Abstract

Existing research is inconsistent regarding the effects of gesture production on narrative recall. Most studies have examined the effects of gesture production during a recall phase, not during encoding, and findings regarding gesture’s effects are mixed. The present study examined whether producing gestures at encoding could benefit an individual’s narrative recall and whether this effect is moderated by verbal memory and spatial ability. This study also investigated whether producing certain types of gesture is most beneficial to recalling details of a narrative. Participants read a narrative aloud while producing their own gestures at pre-specified phrases in the narrative (Instructed Gesture condition), while placing both their hands behind their backs (No Gesture condition) or with no specific instructions regarding gesture (Spontaneous Gesture condition). Participants completed measures of spatial ability and verbal memory. Recall was measured through both free recall, and specific recall questions related to particular phrases in the narrative. Spontaneous gesture production at encoding benefited free recall, while instructed gestures provided the greatest benefit for recall of specific phrases where gesture had been prompted during encoding. Conversely, for recall of specific phrases where gesture had not been prompted during encoding, instructions to either gesture or not gesture suppressed recall for those higher in verbal memory. Finally, producing iconic and deictic gestures provided benefits for narrative recall, whilst beat gestures had no effect. Gestures play an important role in how we encode and subsequently recall information, providing an opportunity to support cognitive capacity.

**Supplementary Information:**

The online version contains supplementary material available at 10.1007/s00426-023-01886-w.

## Introduction

Gestures, movements of an individual’s hands and arms, are pivotal in human communication (McNeill, 1992). Gestures deliver non-verbal information which can emphasize the meaning of concurrent speech and/or take the place of speech (McNeill, 1992). Gestures can influence learning across multiple domains such as mathematics (Cook et al., 2008; Goldin-Meadow et al., 2001; Novack et al., 2014), word acquisition (Iverson & Goldin-Meadow, 2005; Krönke et al., 2013; Rowe et al., 2008), second language (L2) acquisition (Morett, 2017; Sweller et al., 2020), and narrative recall (Bharadwaj et al., 2022; Cameron & Xu, 2011; Dargue & Sweller, 2020b; Vilà‐Giménez & Prieto, 2020). Narrative recall and comprehension, the ability to construct meaning from information in a story, is regarded as a vital milestone in cognitive development due to the skill required to integrate ideas (Paris & Paris, 2003). Due to its potential to enhance learning, it is critical we understand the role gestures can have on narrative recall (Lyle, 2000).

Existing research has found that observing gestures during encoding benefits narrative recall (Dargue & Sweller, 2018a; 2018b; Dargue & Sweller, 2020a; 2020b; Macoun & Sweller, 2016; McNeil et al., 2000). To date, there has been limited investigation into the effect of producing gestures during encoding, with conflicting results across both child and adult samples (Bharadwaj et al., 2022; Cameron & Xu, 2011; Dargue & Sweller, 2020b; Vilà‐Giménez & Prieto, 2020). Given the importance of narratives to learning and teaching, the current study examines the effects of producing gestures during encoding on narrative recall, with a view to clarifying the reasons underlying the previously conflicting results.

Not all gestures are equivalent in either form or function however, and gestures are frequently classified as iconic, deictic, metaphoric or beat (McNeill, 1992). Iconic gestures convey the meaning of co-occurring speech and reflect concrete objects, actions or events (McNeill, 1992). For example, an individual discussing making a phone call might hold a fist up to their ear with their thumb and pinkie finger extended. Deictic or pointing gestures indicate the direction or location of an event or object (McNeill, 1992). For example, pointing to the left when speaking about their house, to signal where it is. Gestures used to present an abstract idea or a metaphor of a concept are termed metaphoric gesture (McNeill, 1992). For example, an individual describing the process of making a decision moves their hands up and down as if weighing up two options on opposite ends of a scale. Finally, beat gestures do not reflect the apparent semantic meaning of co-occurring speech (Alibali et al., 2001). Beat gestures are primarily considered to be rhythmic movements that emphasize a word or phrase, for example when an individual creates a relaxed up and down motion with their hand in conjunction with speech (Alibali et al., 2001).

## Mechanisms underlying the beneficial effects of gesture

There are a number of non-mutually exclusive theories explaining how gesture production can assist narrative recall. We focus here on issues related to cognitive load and the Gesture as Simulated Action framework, as both speak to the perhaps varying benefits of different types of gestures on recall, as well as differences in who might benefit most from gestures.

Depending on the form of the gesture, gestures can provide a distinct visuospatial representation of a spoken message, which can allow for richer or deeper encoding (Goldin-Meadow et al., 2001). As a result, gestures may reduce an individual’s cognitive load by making cognitive resources available for performing other tasks (Goldin-Meadow et al., 2001). Beneficial effects of gestures for cognitive load have been found for children when recalling word and letter lists after solving mathematics equations (Goldin-Meadow et al., 2001) and when recalling unrelated words together with a liquid conservation task (Ping & Goldin-Meadow, 2010). Similar benefits of gesture production have been found for adults, where performing gestures which aligned with the content of concurrent speech resulted in a greater reduction in cognitive load than did gestures which did not align with speech (Cook et al., 2012). In this way, gestures which align with the content of speech, such as iconic gestures, may provide the greatest reduction in an individual’s cognitive load. Iconic gesture production may, therefore, support narrative recall, as performing gestures that align with narrative content may boost the cognitive resources available to effectively recall narrative details.

In the Gesture as Simulated Action framework (GSA), Hostetter and Alibali (2008) propose that gestures reflect motor activity which occurs when individuals think and speak. By creating visible representations of an individual’s thoughts, gestures can assist learning. According to the GSA framework, there are two conditions that must be fulfilled for a person to produce a gesture. The first condition is that individuals’ mental images or simulations about actions they have either performed, observed, or imagined activate their motor system. The second condition is that the motor system activation must go beyond an individual’s threshold or level of resistance for a gesture to be enacted or produced (Hostetter & Alibali, 2008, 2019). According to the GSA framework, gesture production during encoding could support narrative recall as aspects of the story could activate the motor system, such that the activation crosses the gesture threshold. In this way, gestures support learning through the creation of a visible representation of thought.

## Benefits of gesture production for narrative recall

There have been inconsistent findings in the literature examining the effect of producing gestures on narrative recall (Cameron & Xu, 2011; Dargue & Sweller, 2020b; Vilà‐Giménez & Prieto, 2020). Cameron and Xu (2011) found that preschool children who were asked to perform iconic and metaphoric gestures were able to better recall specific details of a narrative than those who were prevented from doing so. In a second experiment, it was found that individuals who performed deictic gestures were able to recall more details than those who were asked to keep their hands still (Cameron & Xu, 2011). Vilà‐Giménez and Prieto (2020) found that individuals who were encouraged to perform beat gestures scored higher on narrative structure and fluency tasks than those who were not allowed to gesture. Conversely, Dargue and Sweller (2020b) found that performing gestures yielded no benefit to narrative recall. Finally, Bharadwaj et al. (2022) found that while performing iconic gestures during encoding benefited adult narrative recall, performing deictic gestures was detrimental to subsequent recall. The unexpected detrimental effect of producing deictic gestures should be interpreted with caution however, due to the low number of deictic gestures produced by participants.

There are a number of potential reasons underlying the inconsistent findings regarding the effects of gesture production on narrative recall. First, with exception of the Bharadwaj et al. (2022) study, previous research has examined the effect of producing gestures during the recall phase of a narrative recall task. If gestures benefit recall through reducing cognitive load, it might be expected that the largest benefits would be associated with producing gestures during the encoding phase, rather than during recall. It is during training, rather than during recall, that an individual might be using their working memory to encode the narrative. Gesturing during encoding of a narrative, such as when reading it aloud, could be expected to activate the motor system at the phrases of the narrative where the gestures are produced. In this way, as outlined by the GSA framework, gesturing while reading a narrative may support narrative recall.

Second, not all narratives are equivalent. The content of individual narratives can vary dramatically, perhaps varying the effects that gesture production might have on recall. For example, a typical narrative like the ones used in literature noted above would require participants to gain a comprehensive understanding of characters, details, and events. Narratives including spatial content, however, can have different characteristics: such narratives incorporate route directions, which require the processing of verbal, visual and motor information (Austin et al., 2018). Going beyond simple route directions, such narratives include both content and structure involved in narratives (see Parrill et al., 2018, for an example of narrative structure), as well as spatial content such as directions. Following the GSA framework, if gesture production reflects motor activity, gestures may be particularly beneficial when performed in conjunction with tasks involving spatial content, such as narratives with explicit inclusion of spatial information such as directions, movements and locations.

Finally, few of the above studies examined the effects of gesture production for adults. Differences in language development may contribute to the variability in findings, as if indeed gestures relieve cognitive load, perhaps children will experience more benefits of gesture production than adults. The current study, therefore, examines the effects of the production of different types of gestures on recall of a narrative incorporating spatial content by adults. It is possible that not all participants will benefit equally from gesture production, however. We turn now to a consideration of individual differences that may affect the beneficial effects of gesture production on narrative recall.

## Individual differences

Individual differences could be responsible for moderating the extent of benefit on narrative recall gained from producing gestures (McKern et al., 2021). Here, we examine verbal memory, which is relevant for written narratives, and spatial ability, which is relevant when narratives include spatial content.

### Gesture and verbal memory

As previously mentioned, producing gestures is thought to decrease an individual’s cognitive load and free up working memory resources (Goldin-Meadow, 2001; Cook et al., 2012). It is likely, therefore, that performing gestures can aid the management of verbal working memory demands, namely demands placed on an individual’s memory for words or language-based tasks (Wagner et al., 2004). Gillespie et al. (2014) investigated whether individuals with differing levels of verbal memory would gesture to varying degrees (Gillespie et al., 2014). Participants were asked to watch cartoons and then describe the actions of people in the videos, followed by several measures assessing verbal memory. Individuals who scored low on measures of verbal working memory were more likely to perform gestures when describing actions than those with higher verbal working memory. Further, Pyers et al. (2021) examined the effect of inhibiting gestures for individuals attempting to resolve tip of the tongue words. Gesture production was found to interact with verbal, but not spatial, short term memory: inhibiting gestures was detrimental to those with lower verbal short term memory. It is likely, therefore, that producing gestures at encoding could benefit individuals who possess lower verbal memory more than individuals with higher verbal memory through a reduction in cognitive load, aiding subsequent task performance.

### Gesture and spatial ability

Spatial ability can be defined as an individual’s capability to mentally manipulate stimuli, recognize how an object is located in reference to another and identify the direction of an object in space (Austin et al., 2018). Consequently, spatial communication tasks can place heavy cognitive demands on an individual’s resources. Individuals who possess high spatial ability have been found to perform better on spatial route direction tasks (Austin et al., 2018). Austin and Sweller (2018) investigated the types of gestures performed when relaying route directions. Children and adults were taken on a short walk around their preschool or university and asked to describe two routes: the novel route that they were taken on as well as the route they take from home (Austin & Sweller, 2018). Children and adults primarily used deictic and iconic gestures to convey route directions, consistent with prior research that showed iconic gestures preserve spatial information (Perniss & Özyürek, 2015). The use of gesture to convey spatial information suggests that producing gestures could aid route recall, and it is possible that those who experience difficulty in performing spatial tasks could gain more benefit from gesturing. That is, individuals with lower spatial ability could receive more benefit in recalling a narrative involving spatial content from producing gestures at encoding than those with higher spatial ability, as they find the task more cognitively demanding.

## Present study

The present study aimed to examine whether producing gestures during narrative encoding benefits recall. Given that processing narratives with spatial content places high demands on cognitive resources, we hypothesized that across gesture types, those who were instructed to gesture during encoding would remember more of the narrative than those who either received no instruction regarding gesture or were prevented from gesturing, and those who received no instruction regarding gesture would in turn remember more of the narrative than those prevented from gesturing. We primarily expected this effect for specific phrases in the narrative where participants produced a gesture at encoding, rather than for phrases where they did not produce a gesture at encoding.

Verbal memory and spatial ability may be important for processing narrative content, as well as determining the degree of benefit derived from producing gesture at encoding. The present study aimed to examine whether the effect of producing gestures on narrative recall is moderated by verbal memory and spatial ability. We expected that individuals with higher levels of verbal memory would recall more of the narrative, and those with higher levels of spatial ability would also recall more. We anticipated a moderating effect, such that the beneficial effects of gesture production at encoding would become weaker as verbal memory capacity and / or spatial ability increased.

The final aim of this study was to investigate whether producing different types of gestures would influence recall of specific phrases where participants produced each gesture type. Given the unexpected findings by Bharadwaj et al., (2022), although production of all gesture types was of interest, deictic gestures were a particular focus, and the current study aimed to elicit such gestures by participants during encoding. We expected participants to recall more for specific phrases of the narrative where they produced a gesture at encoding than for phrases where they did not produce a gesture at encoding.

## Method

### Ethics approval and participants

The Ethics application for this study was approved by the Macquarie University Human Research Ethics Committee (Reference Code: 52,021,965,728,918) prior to commencement. After obtaining ethics approval, first year introductory psychology and second year cognitive psychology students were recruited through an advertisement displayed on the Macquarie University Participant Pool website. All participants were required to be fluent in English, have corrected to normal vision and corrected to normal hearing for eligibility. An a priori power analysis in G*power version 3.1.9.7 (Faul et al., 2007) was calculated based on the effect size obtained for the interaction by Bharadwaj et al. (2022) of *η*_*p*_^*2*^ = 0.09. For power of 0.8, 101 participants were needed. A total of 99 students were recruited for the study. Nine students were excluded from final analyses due to experiment piloting, experimenter error, or technical difficulties. The final sample consisted of 90 students (26 males, 64 females) ranging from 18 to 32 years of age (*M* = 20.91, *SD* = 3.33).

### Experimental design

Participants were randomly allocated into one of three between-subjects gesture conditions, with 30 participants in each of the Instructed Gesture, No Gesture and Spontaneous Gesture conditions. Verbal memory and spatial ability were measured as continuous predictors. The dependent variables were free recall and specific recall of questions related to individual phrases in the narrative.

## Materials

### Narrative and recall questions

The primary stimulus was a 453-word fictional narrative about a young snail’s journey to school (see Appendix A for the complete narrative). Participants were asked to read the narrative out loud whilst standing up at a comfortable distance from their laptop or computer screen, to enable the experimenter to see their face and hands while reading. Fourteen phrases in the narrative were written in bold and the remainder were not bolded. Participants in the Instructed Gesture Condition were asked to produce gestures at every bolded phrase during reading. The bolded text was selected to elicit either iconic or deictic gestures. For example, the phrase “grabbed his snowboard” is likely to elicit an iconic gesture, while the phrase “his left” is likely to elicit a deictic gesture. The form of gestures that participants should produce was not stipulated. Participants in the No Gesture Condition were asked to place their hands behind their backs while reading, to prevent inadvertent gesture production. Participants in the Spontaneous Gesture Condition were given no specific instructions regarding gesture production.

The response items included one free recall question and 21 specific recall questions (see Appendix B for recall questions). The participants were initially asked the free recall question, asking them to tell the experimenter everything they remembered from the narrative. Participants were then asked the specific questions which related to the fourteen bolded phrases in the narrative and to seven non-bolded phrases. When a participant responded correctly to a specific question, they were asked the next specific question. If a participant answered a specific question incorrectly or could not provide a response, they were asked a binary response option forced choice question. The order of presentation of specific recall questions was randomized through an online number generator (https://www.randomizer.org/) separately for each participant.

### Verbal memory task

Participants’ verbal memory was measured using the Rey Auditory Verbal Learning Test (RAVLT; Rey, 1941). This test consists of five consecutive trials where participants learn a list of words, an interference trial using a different list, an immediate recall trial, a delayed recall trial and a recognition trial. The recognition trial was not of interest for the purposes of the current study and was not administered to participants. The learning and interference trials assess an individual’s acquisition as opposed to memory retention capabilities and are not discussed further here (Ferreira Correia & Campagna Osorio, 2014). Only the immediate and delayed recall trials were examined in the current study. Each trial of the RAVLT was scored out of 15, with participants gaining one point for every correct word remembered (see Strauss, 2006). Internal reliability of this test is 0.90 (Van Den Burg & Kingma, 1999).

### Spatial ability task

Participants’ spatial ability was measured through the Santa Barbara Sense of Direction Scale (SBSOD) (Hegarty et al., 2002), comprised of 15 self-report statements. These self-report statements assess an individual’s spatial abilities, navigational abilities, preferences, and experiences, for example “I am very good at giving directions”. This scale was chosen to measure spatial ability in the current study as it explicitly taps into broad spatial and navigational abilities including directions. Given the narrative used in the current study involves directional content such as “turned right” and “his left”, a scale measuring directional ability rather than the mental rotation tasks sometimes used in previous studies examining spatial ability and gesture (e.g., Parrill et al., 2018) was deemed most appropriate. Items are measured on a seven-point Likert scale, where participants rate their strength of agreement to the statements, ranging from (1) strongly agree to (7) strongly disagree. Total scores were created by summing all items and ranged between 15 and 105, with a lower score representing higher perceived spatial ability. Internal reliability of this test is 0.88.

### Recording and administration

As data collection occurred during the COVID-19 pandemic, the experiment was conducted online using the Zoom platform. Participants were required to have access to a working webcam and microphone on either a laptop or desktop computer. Prior to commencement of the study, participants were informed that video in addition to audio recordings would be used for statistical analysis purposes and all provided consent. Participants were told that they could stop the experiment at any time if they did not feel comfortable with being recorded.

## Procedure

The study took approximately 45 min per participant. After providing informed consent via Qualtrics (online survey platform), participants completed a demographic survey. Participants were then asked to verbally confirm consent to be audio and video recorded for the experiment. The experimenter then shared his screen, and participants were asked to read the narrative aloud according to their condition allocation (Instructed Gesture, No Gesture, Spontaneous Gesture). No opportunity was given to participants to read the narrative to themselves before they began reading aloud. Participants then completed the SBSOD scale via Qualtrics. The SBSOD doubled as a filler task, to prevent ceiling performance. Following administration of the SBSOD, participants were asked the free recall question, followed by the specific questions relating to the bolded and non-bolded phrases. The first five learning trials of the RAVLT were then administered, along with the interference trial, followed by the immediate recall trial. Participants then received a link to an online version of Tetris (https://n-blox.com/) which acted as a 20-min filler task before the final RAVLT delayed recall trial. Although Tetris involves mental rotation, it does not incorporate any verbal element (or any spatial directional information such as the words “left”, “right” etc.), and was therefore deemed an appropriate filler task for a study on narrative recall. All participants were informed when there were ten minutes left and five minutes left of playing Tetris. Once the final RAVLT trial had been completed, participants were asked what they thought the overarching purpose of the study was. A majority of the participants stated that they believed the study was about memory and more specifically retaining memory after being distracted. Finally, participants were debriefed by the experimenter and informed of the purpose of the study. Participants were thanked for their participation and received course credit.

### Scoring and coding

The scoring method used for the present study was based on that of Dargue and Sweller (2018b, 2020b). The speech and gestures produced by participants during encoding and recall were noted and coded in the software program ELAN (The Language Archive, 2021). Filler terms (e.g., “umm”) were included as parts of speech. The definition of gestures in this study included body movements used by participants to highlight a portion of the narrative (e.g., moving their head down to signal “Fred looked down”). Incidental hand movements (e.g., adjusting hair, scratching) were not coded. Gestures were coded according to one of the four categories (i.e., iconic, deictic, metaphoric, beat) outlined by McNeill (1992) and defined above. It should be noted that in relaying spatial information, gestures may include both iconic and deictic content (see Austin & Sweller, 2018). To maintain consistency with Bharadwaj et al. (2022) however, the current study coded gestures as only iconic or only deictic, and a code was allocated according to whichever category each gesture more clearly aligned with.

In the free recall phase, one point for correct free recall was given when participants provided a response which displayed an accurate understanding of the content. Conversely, no points were given if an incorrect item or event was mentioned, or if an item or event was not mentioned at all. The maximum free recall score participants could receive was 190 points for correctly remembering all details in the narrative. For the specific recall questions, participants were awarded 2 points for every correct answer to a question, to a maximum of 42 points. If a participant gave an incorrect response to a specific question but correctly answered the related, forced choice follow-up question, they received one point for that item. If they were unable to provide a correct answer in either the initial specific or forced choice question, they received zero points for that question.

Consistent with previous research in the field of narrative recall and gesture studies (e.g., Dargue & Sweller, 2018b; Vilà‐Giménez & Prieto, 2020), a second coder independently coded a random 20% of participants’ encoding and recall trials. Single measure intra-class correlations were calculated using an absolute agreement model. For gestures produced at encoding, iconic ICC = 0.85, *p* < 0.001; deictic ICC = 0.95, *p* < 0.001; beat ICC = 0.69, *p* < 0.001. No metaphoric gestures were produced by participants in the sample chosen for inter-rater reliability coding. At recall, free recall ICC = 0.87, *p* < 0.001 and specific questions ICC = 0.98, *p* < 0.001.

### Analysis plan

Stata 17 (StataCorp, 2021) was used for all analyses. Both continuous predictors (verbal memory and spatial ability) were mean centered prior to analysis. ANCOVAs with interactions were used to assess the effects of gesture condition, verbal memory and spatial ability on recall, with gesture condition as the between-subjects factor, and each of verbal memory and spatial ability as continuous predictors. The family-wise error rate was controlled at 0.05, with follow-up tests Bonferroni adjusted. The extent to which recall was improved for questions in the narrative relating to phrases where participants gestured during encoding compared to when they did not gesture was assessed with a mixed effects ordinal logistic regression due to the nested nature of the data, with recall of specific items in the narrative nested within participants. Gestures performed during encoding were matched to the corresponding specific question at test. Although the expected relationship should hold for free recall as well as for recall of specific questions, it is less clear for free recall than for recall of specific questions exactly which point in the narrative participants are referring to. Direct matching of gestures performed during encoding and free recall is therefore less reliable than matching for specific questions. This aim was therefore only addressed in relation to specific recall questions.

For analyses in which the assumption of normality of residuals was violated, bootstrapping was performed. After bootstrapping, Stata reports the overall effects of categorical variables as chi squared statistics, and the overall effects of continuous predictors as z statistics. Pairwise comparisons for levels of categorical variables are similarly reported as z statistics and are reported as such below. No bootstrapping was performed for analyses where the assumption was not violated, and results are reported as *F* statistics.

## Results

### Effects of gesture condition, verbal memory, and spatial ability on recall

A between-subjects ANCOVA predicting total free recall score from immediate verbal memory (*M* = 11.52, *SD* = 2.79), spatial ability (*M* = 61.59, *SD* = 6.02), gesture condition, and the interactions between each covariate and gesture condition was conducted. See Table 1 for means and standard deviations of free recall by gesture condition. A significant main effect of gesture condition on free recall was found, *χ*^*2*^(2) = 11.99, *p* = 0.003, *η*_*p*_^*2*^ = 0.12, such that participants in the Spontaneous Gesture condition recalled more than individuals in the Instructed Gesture condition, *z* = 3.45, *p* = 0.001, *η*_*p*_^*2*^ = 0.12. No other pairwise comparisons, and no other main effects or interactions, were significant (all *p* > 0.05). The same pattern of results was obtained when using delayed, rather than immediate, verbal memory (analyses available from authors on request).

**Table 1:** Mean and standard deviation free recall and specific questions scores for gestured and non-gestured phrases, by gesture condition

	Gesture condition					
Measure	Instructed gesture		No gesture		Spontaneous gesture	
	*M*	*SD*	*M*	*SD*	*M*	*SD*
Free recall	17.13	10.61	21.60	11.94	27.20	11.22
Specific questions for gesture phrases	14.27	2.85	12.47	2.30	14.07	3.40
Specific questions for non-gesture phrases	6.13	1.94	6.37	1.67	7.03	2.30

A between-subjects ANCOVA predicting the total score of specific questions related to narrative phrases where participants were prompted to gesture during encoding, from gesture condition, immediate verbal memory, spatial ability and the interactions between gesture condition and each continuous predictor was conducted (see Table 1). A significant main effect of gesture condition was found, *F*(2, 81) = 4.10, *p* = 0.02, *η*_*p*_^*2*^ = 0.09. While there was no significant difference between the Instructed and Spontaneous Gesture conditions, *F*(1, 81) = 0.22, *p* = 0.64, *η*_*p*_^*2*^ = 0.003, participants in the Instructed Gesture condition recalled significantly more than those in the No Gesture condition, *F*(1,81) = 7.18, *p* = 0.01, *η*_*p*_^*2*^ = 0.08. Finally, participants in the Spontaneous Gesture condition recalled more gesture items than those in the No Gesture condition, *F*(1,81) = 5.06, *p* = 0.03, *η*_*p*_^*2*^ = 0.06 (non-significant following Bonferroni adjustment). There was a significant main effect of immediate verbal memory on specific questions relating to gesture phrases, suggesting a positive relationship between immediate verbal memory and recall, *F*(1,81) = 5.67, *p* = 0.02, *η*_*p*_^*2*^ = 0.07. There were no other significant main effects or interactions (all *p* > 0.05). Again, the same pattern of results held when using delayed, rather than immediate, verbal memory.

**Fig. 1:**
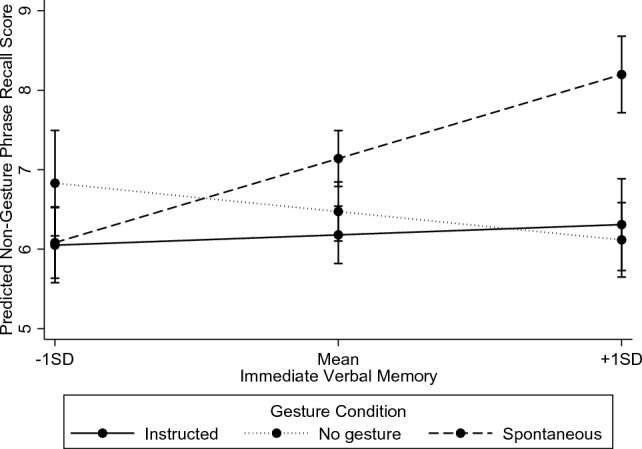
Interaction effect between gesture and immediate verbal memory on the recall of specific questions related to non-gesture phrases

A between-subjects ANCOVA predicting total score on specific questions related to narrative phrases where gesturing was not prompted during encoding, from gesture condition, immediate verbal memory, spatial ability and the interactions between gesture condition and each continuous predictor was conducted (see Table 1). There were no significant main effects of gesture condition, immediate verbal memory or spatial ability on recall, and no significant interaction between gesture condition and spatial ability (both *p* > 0.05). However, there was a significant interaction between gesture condition and immediate verbal memory, *F*(2,81) = 4.08, *p* = 0.02, *η*_*p*_^*2*^ = 0.09 (see Fig. 1).

Follow-up tests of simple effects comparing the Instructed Gesture, No Gesture and Spontaneous Gesture conditions were run at one standard deviation below the mean, the mean and one standard deviation above the mean of immediate verbal memory. At one standard deviation below the mean, and at the mean, of immediate verbal memory, there were no significant differences between conditions (all *p* > 0.017). At one standard deviation above the mean of immediate verbal memory, however, there were significant differences between the Instructed and Spontaneous Gesture conditions, *F*(1.81) = 6.39, *p* = 0.01, *η*_*p*_^*2*^ = 0.01 as well as the No Gesture and Spontaneous Gesture condition, *F*(1.81) = 9.69, *p* = 0.003, *η*_*p*_^*2*^ = 0.11, such that the Spontaneous Gesture condition performed significantly better than each of the Instructed and No Gesture conditions. There was no significant difference between the Instructed and No Gesture conditions, *F* (1.81) = 0.07, *p* = 0.80, *η*_*p*_^*2*^ < 0.001. When replicating this analysis with delayed, rather than immediate verbal memory, the interaction between gesture condition and delayed verbal memory became non-significant, at *p* = 0.08. All other results mirrored those found with immediate verbal memory.

### The effect of production of different gesture types on narrative recall

A mixed effects ordinal logistic regression was conducted to investigate whether production of certain types of gestures during encoding enhanced recall of specific questions at test (see Table 2 for frequencies by recall score).

**Table 2:** Frequencies and percentages of scores across specific questions

Specific question recall score	Frequency	Percentage
0	451	23.86
1	1068	56.51
2	371	19.63

Predictors were dummy codes indicating whether an iconic, deictic or beat gesture was performed by participants during encoding at the phrase which corresponded to each specific question (see Table 3 for gesture production frequencies and percentages).

**Table 3:** Frequencies and percentages of gestures produced during encoding, by gesture type

	Gesture type					
Gesture performed	Iconic		Deictic		Beat	
	Frequency	Percentage	Frequency	Percentage	Frequency	Percentage
Yes	228	12.06	147	7.78	34	1.80
No	1662	87.94	1743	92.22	1856	98.20

The overall regression model was not significant, *Wald χ*^*2*^(3) = 6.76, *p* = 0.08. A significant positive effect of iconic gestures on recall was found however, *β* = 0.31, *SE* = 0.16, *z* = 1.99, *p* = 0.047, suggesting that participants who performed an iconic gesture during encoding were more likely to obtain a higher score on corresponding specific questions at test compared to individuals who did not perform an iconic gesture during encoding. There was also a significant positive effect of deictic gestures, *β* = 0.38, *SE* = 0.19, *z* = 1.99, *p* = 0.047, suggesting that participants who made a deictic gesture during encoding were more likely to obtain a higher recall score on corresponding specific questions at test than individuals who did not perform a deictic gesture during encoding. There was no significant effect of beat gesture production on recall, *β* = 0.36, *SE* = 0.34, *z* = 1.05, *p* = 0.29.

## Discussion

The present study examined the effect of producing gestures during encoding on narrative recall. Participants freely recalled more of the narrative when allowed the opportunity to spontaneously gesture compared with being instructed to gesture. Conversely, instructions to gesture resulted in the highest recall of specific items in the narrative at which gesturing was prompted. While for phrases at which gesturing was not prompted during encoding the expected positive relationship between verbal memory and recall was found for those in the spontaneous gesture condition, for those instructed to either gesture or not gesture, recall performance was suppressed for those higher in verbal memory. Finally, both iconic and deictic gesture production improved recall, while beat gestures had no effect.

## Effect of gesture condition on narrative recall

Across free recall and specific recall of gesture phrases, it was expected that participants in the Instructed Gesture condition would perform better than those in the Spontaneous and No Gesture conditions. Unexpectedly, participants in the Spontaneous Gesture condition performed significantly better than participants in the Instructed Gesture condition on free recall. On the surface, this finding appears inconsistent with the idea that the act of producing gestures leads to lessening of cognitive demands and freeing up resources for subsequent tasks such as recall (Cook et al., 2012; Goldin-Meadow, 2001). It is possible, however, that stipulating where participants should gesture placed an additional cognitive load itself. Rather than producing gestures naturally, participants had to consciously think of what gestures to produce when they were prompted to do so. Additionally, as participants in the Instructed Gesture condition were asked to gesture at specific phrases of the narrative, they may have interpreted this instruction as implying they should not gesture for other phrases. This could similarly have increased cognitive demands, as participants were essentially asked to perform another task of suppressing gestures in some parts of the narrative, and producing them in others (Overoye & Wilson, 2020).

Conversely, and consistent with expectations, a significant effect of gesture condition on recall of specific questions related to gesture phrases was found, but no significant main effect of gesture condition on recall of specific questions related to non-gesture phrases. For gesture phrases, participants in the Instructed Gesture condition performed significantly better than those in the No Gesture condition, consistent with the theory that gesture production lightens cognitive load (Cook et al., 2012; Goldin-Meadow, 2001). This finding similarly suggests that performing gestures during encoding can aid the recall of the specific details related to when the gesture was produced, rather than of recall overall, or of details where gestures were not produced.

## Individual differences

A positive relationship was expected between verbal memory and recall performance. In addition, the effect of gesture production on recall was expected to be moderated by verbal memory. Unexpectedly, there was a significant interaction between gesture and verbal memory only for specific recall of non-gesture phrases, and not in the expected direction. Rather, while the recall of those low and at the mean of non-verbal memory did not differ between gesture conditions, for those high on non-verbal memory, those in the Spontaneous Gesture condition outperformed those in either other condition. In other words, while the expected positive relationship between verbal memory ability and recall was seen for those in the Spontaneous Gesture condition, instructions either to gesture or to not gesture appear to have suppressed performance for those higher in non-verbal memory. As noted above, it is possible that those in the Instructed Gesture condition interpreted their instructions to gesture at specific phrases to mean they should not gesture at other phrases, inhibiting recall of these non-gesture items.

As gesture and speech form an integrated system (McNeill, 1992), asking participants not to gesture may have interfered with that system. The integrated gesture-speech system may have been impaired by the inability to use gestures, bringing the performance of those higher on verbal memory down to levels similar to those lower on non-verbal memory. As the Spontaneous Gesture condition is the only one in which participants received no instructions regarding gesture, and therefore experienced no interference to the gesture-speech system, that is the only condition on which the expected relationship between verbal memory abilities and recall is seen. Those with higher verbal memory abilities may have a more efficient gesture–speech system, and so the interference with that system is most marked for those participants.

Across free recall and specific recall of gesture phrases, it was expected both that spatial ability would be positively related to recall, and that participants with poorer levels of spatial ability would receive more benefit from producing gestures at encoding than those with higher levels of verbal memory. Contrary to expectations, no effects of spatial ability were seen. It is possible that although the narrative contained spatial content, designed to facilitate deictic gesture production, it did not tap into broader spatial skills. In other words, the SBSOD scale, which is designed to measure these broader spatial skills, may not have measured a skill directly needed for this particular narrative.

## Effects of different gesture types on recall of individual phrases

It was expected that participants would recall more for specific phrases of the narrative where they produced a gesture at encoding, compared to phrases where they did not. Indeed, both iconic and deictic gestures positively influenced recall, such that participants had an increased likelihood of gaining a higher recall score for the phrases in the narrative where iconic or deictic gestures were produced, compared to phrases where they were not produced. There was no effect of beat gestures on narrative recall.

These findings suggest that producing iconic gestures at encoding can positively influence recall, consistent with previous literature which demonstrated that iconic gestures are beneficial for learning (Cameron & Xu, 2011; Ping & Goldin-Meadow, 2010). In this study, the production of iconic gestures may have lessened the cognitive demand placed on verbal working memory to process the content of the narrative and therefore allowed greater encoding. This is consistent with the theory that gesture lightens cognitive load, as it proposes that gestures which align with the content of concurrent speech are ideal for reducing cognitive demands (Cook et al., 2012; Goldin-Meadow, 2001). This finding can also be explained using the GSA framework, as participants may have produced mental images whilst processing the narrative, which could lead to performance of meaningful gestures that benefit encoding.

These findings also suggest that producing deictic gestures at encoding can positively influence recall, contrary to the findings of Bharadwaj et al., (2022). As previously noted, the unexpected finding of deictic gestures having a negative effect of recall in the study conducted by Bharadwaj et al., (2022) should be interpreted with caution as only few were produced in total. In the current study, the production of deictic gestures, similar to iconic gestures, may have lessened the cognitive demand placed on the verbal working memory to process the content of the narrative and therefore allowed greater encoding, again consistent with the idea that gesture lightens cognitive load (Cook et al., 2012; Goldin-Meadow, 2001), and with the GSA framework.

Conversely, beat gesture production did not benefit narrative recall, contrary to the study by Vilà-Giménez and Prieto (2020). There are important methodological differences between the 2020 and current studies, however. In the 2020 study, participants were first asked to watch videos and pay attention to the beat gestures used by the narrator who was telling the stories. As participants in the experimental group were encouraged to use beat gestures in the same way as the narrator to retell a story, the fact that participants firstly observed beat gestures being used could have influenced their ability to perform on the narrative tasks. In the current study however, no modeling of beat gestures was provided, and the phrases on which gestures were purposefully elicited were chosen to prompt iconic or deictic, rather than beat, gestures.

## Limitations and future research suggestions

As mentioned previously, unexpected findings such as the direction of the gesture condition main effect on free recall and the significant interaction between gesture and verbal memory on non-gesture phrases, could have resulted from stipulating for participants precisely where they should gesture. Although this manipulation was implemented to ensure participants produced sufficient deictic gestures, it may have had unintended consequences on both participants’ performance of gestures on other phrases in the narrative, as well as their overall cognitive load resulting from creating gestures for pre-specified phrases, rather than phrases where individually they would naturally have gestured. Future research might use a narrative which is designed to elicit deictic gestures, as with the current narrative, but not stipulate where to produce gestures, mirroring Bharadwaj et al., (2022).

It is notable that as the present study did not directly assess the cognitive load placed on working memory, it cannot be assumed that gesture production alleviated cognitive load. Future studies should examine whether load is lightened through scales such as the Cognitive Load Questionnaire (Leppink et al., 2014), or with a dual-task paradigm (Wagner et al., 2004).

## Conclusion

The primary aim of the present study was to examine whether producing gestures during encoding would positively influence narrative recall. Additionally, it was investigated whether individual differences in verbal memory and spatial ability could influence the amount of benefit individuals receive from gestures. Gesture production was found to inconsistently influence narrative recall, with spontaneous gesture production benefiting free recall and instructed gesture being of most benefit for recall where gesturing had been indicated during encoding. Furthermore, for recall of phrases where gesture was not indicated during encoding, instructions to either gesture or not gesture were detrimental to recall for those with higher levels of verbal memory. Finally, iconic and deictic gestures positively influenced narrative recall, whilst beat gestures had no effect. Results are in line with the idea that meaningful gestures can lighten cognitive load. These findings highlight the importance of examining not just whether learners perform gestures, but what types of gestures they perform, as well as the individual characteristics of each learner. Through the right combination of targeted gesture instruction to differing individuals, gestures can be leveraged to enhance learner recall.

### Supplementary Information

Below is the link to the electronic supplementary material.

Supplementary file1 (DOCX 18 KB)

## Data Availability

De-identified datasets are available from the corresponding author on request.
