# Supplementary material for: Gesture production at encoding supports narrative recall
Source: Psychol Res. 2023 Oct 19;88(2):535–46. doi: 10.1007/s00426-023-01886-w (PMC10858132; doi:10.1007/s00426-023-01886-w)
Supplement: Supplementary file 1 — Supplementary file1 (DOCX 18 KB) [file 426_2023_1886_MOESM1_ESM.docx]

# Appendix A: Narrative

# The Long Journey to School

It was a cold, frosty morning and Fred through his window saw that every single car outside was covered in white. The inch-high snail then **looked down** because he had to go to school in this weather. Fred was adamant that he couldn’t attended and earnestly **pleaded to his mother**. However, his pleas were to no avail and so he began to get ready for school. It was going to be a difficult morning.

Fred urgently got his backpack, snowboard, jacket and then **ran outside from his house** because he could not be late to school. However, he abruptly stopped as **hail from above** kept on falling everywhere. Soon, the hail became a truckload of snow and Fred simply had to **walk through it**. After walking for a while, he had arrived at the end of the road. Fred then **turned right** and could finally see his school which was half a mile away, down a steep hill. Fred **began to think** how he was going to make it that far. Suddenly, the school started moving towards him at a rapid pace. The path became clear. Ice.

He then **grabbed his snowboard** from behind and began to slide down the hill. "AAAAAAAARGGGGHHHH!" Fred screamed.

He slid past the houses to **his left**. He then slid past the bank.

This wasn’t scary anymore. It was fun. ‘Whooooooooo!’ Fred shouted.

He then even slid past the library to **his right**.

Fred had never travelled so fast in his life. He was ecstatic and felt like a jet pilot in the sky.

However, that changed when he **saw the playground over there** sprinkled in white. Salt can kill snails like Fred and he couldn’t slow down. He could not stop either and frankly he **does not know what to do**. He did not want to die though, so his only choice was to steer and thus Fred aimed for the edge of the curb when it sloped up. Soon, he shot into the air and scraped the top of the fence. Now, he could finally see the closed school doors. Fred then shut both of his eyes hoping that wouldn’t splat into it.

The wind began to howl and then he heard a defining, loud noise. DING! DING! DING!

It was the school bell!

Fred knew that if the school bell rang then the doors would be opened. As soon as the principal opened the doors, Fred zoomed past her and then skidded across the floor. After he got up, he then went into the classroom which he saw was **behind him**. He was cold. He was confused. He was dizzy. He was right on time and **commended himself** for not being late to school.

# Appendix B: Interview Script

Now, I am going to ask you some questions about the story you read earlier and if you don’t know the answers you can just guess.

**Free Recall Question**: First, tell me everything you remember about the story you read earlier.

I am now going to ask you a few more questions about the story you just read. The questions I am going to ask won’t necessarily be in the same order as what you read in the narrative.

*Questions From Gesture Phrases*

Q1) What was the first thing Fred did after he saw every car covered in white?

- 1. Did he look down or get ready?

Q2) What did Fred do directly after arriving at the end of the road?

1. Did Fred simply stand or turn right?

Q3) Why did Fred abruptly stop after getting out of his house?

1. Was it because he slipped or because hail from above fell everywhere?

Q4) What direction was the library when Fred was sliding down the hill?

1. Was it straight ahead or to the right?

Q5) What direction were the houses when Fred was sliding down the hill?

1. Were they to his left or right?

Q6) What direction was the classroom to Fred?

1. Was it to his left or behind him?

Q7) Why did Fred’s feelings change so suddenly after he slid past the library?

1. Was it because he saw the playground over there or because he couldn’t stop?

Q8) What did Fred do to try and avoid school?

1. Did he make a fit or plea to his mother?

Q9) What did Fred do when he saw the truckload of snow?

1. Did he give up or walk through it?

Q10) What did Fred do because he wanted to avoid being late to school?

1. Did he run outside from his house or get ready incredibly early?

Q11) What did Fred grab in order to slide down the hill?

1. Did he grab his jacket or his snowboard?

Q12) What did Fred do straight after seeing his school when it was half a mile away?

1. Did he go back home or think about how he was going to get there?

Q13) What did Fred do when he arrived at school on time?

1. Did Fred sigh in relief or commend himself?

Q14) What was Fred’s initial reaction when he could not stop sliding and slow down?

1. Did he come up with a plan being fully aware or did he frankly not know what to do?

*Questions From Non-Gesture Phrases*

Q1) Why did Fred shut his eyes when he was near his destination?

1. Was it because he wanted to avoid splatting into the doors of the school or because he heard a loud noise?

Q2) What did Fred do when his pleas were to no avail?

1. Did he make a tantrum or begin to get ready for school?

Q3) What made the path become clear?

1. Was it the fact that Fred’s school began to move towards him or that he cleaned it up?

Q4) What did Fred do as soon as the principal opened the school door?

1. Did he walk past the principal or zoom past the principal?

Q5) When could Fred see the closed school doors right in front of him?

1. Was it when he reached the end of the road or after he scraped the top of the fence?

Q6) When did Fred hear the defining, loud noise?

1. After he had entered school building or before when the wind was howling?

Q7) What did Fred know as soon as the bell rang?

1. Did he know that the bell ringing signaled the opening of the school doors or the closing of the school door?
